# Characterization, Bioactivity and Application of Chitosan-Based Nanoparticles in a Food Emulsion Model

**DOI:** 10.3390/polym13193331

**Published:** 2021-09-29

**Authors:** Karina Oliveira Lima, Cristian Mauricio Barreto Pinilla, Ailén Alemán, M. Elvira López-Caballero, M. Carmen Gómez-Guillén, Pilar Montero, Carlos Prentice

**Affiliations:** 1 Laboratory of Food Technology, School of Chemistry and Food, Federal University of Rio Grande (FURG), Rio Grande, RS 96203-900, Brazil; karinah_ol@hotmail.com (K.O.L.); dqmprent@furg.br (C.P.); 2 Laboratory of Applied Microbiology and Biochemistry, Institute of Food Science and Technology (ICTA), Federal University of Rio Grande do Sul (UFRGS), Porto Alegre, RS 90650-001, Brazil; mauricio.barreto@ufrgs.br; 3 Institute of Food Science, Technology and Nutrition (ICTAN-CSIC), 28040 Madrid, Spain; ailen@ictan.csic.es (A.A.); cgomez@ictan.csic.es (M.C.G.-G.)

**Keywords:** chitosan, polymeric nanoparticles, fish protein hydrolysate, delivery system, bioactivity

## Abstract

In this study, chitosan nanoparticles (CNPs) were prepared by the ionic gelation technique with tripolyphosphate (TPP), and the effect of CNP composition and physicochemical characteristics were evaluated. After the synthesis optimization, CNPs were used as carriers for a fish protein hydrolysate (FPH) with bioactive properties (CNPH). The physicochemical characteristics, antioxidant capacity and antimicrobial, antihypertensive and emulsifier properties of unloaded and loaded CNPs in a food system model were studied. CNPH showed a uniform particle distribution, size ~200 nm, high stability (zeta potential around 30 mV), radical scavenging activity and increased antimicrobial activity against *Staphylococcus aureus*, *Shigella sonnei* and *Aeromonas hydrophila*. Additionally, CNPH showed an angiotensin I-converting enzyme (ACE)-inhibitory activity of 63.6% and, when added to a food emulsion model, this system containing CNPs, with or without FHP, exhibited improved food emulsion stability. Thus, CNPs were able to carry the FPH while maintaining their bioactive properties and can be an alternative to the delivery of bioactive peptides with potential as an emulsion stabilizer for food applications.

## 1. Introduction

Chitosan (C) is a natural linear polycationic heteropolysaccharide composed of β-1,4-linked d-glucosamine and N-acetyl-d-glucosamine obtained by the deacetylation of its parent polymer chitin, the second most abundant natural polymer in nature after cellulose [1]. Chitosan exhibits exceptional biological characteristics for food applications, such as biocompatibility, biodegradability, and nontoxicity [2]. It also possesses mucoadhesive properties and exerts antimicrobial activity against a broad spectrum of microorganisms including Gram-positive and Gram-negative bacteria, filamentous fungi and yeast [3] and can also be used as blends with other polymers and natural compounds to obtain bioactive edible films for foods [4]. Chitosan can be physically modified by using a suitable nanoparticle (NP) synthesis procedure to improve its properties and increase its industrial applications [5]. However, the search and diversification of interesting applications of NP as a food ingredient remains a challenge.

Among the diverse properties of C, increasing interest has been given to the use of C and chitosan nanoparticles (CNPs) in the stabilization and production of emulsions such as pickering emulsions [6,7]. Several studies describe the stabilization of O/W (oil/water) emulsions by CNPs, in which the CNPs are adsorbed onto the oil surface through interactions with an added anionic surfactant or protein [8,9]. These properties of CNPs in O/W emulsions not only create the desirable textural attributes, but also can stabilize emulsion droplets against gravitational separation [10]. In most cases, CNPs act as an emulsion stabilizer by forming an interfacial complex with absorbed surface-active agents (i.e., anionic surfactants or proteins), acting mainly by a steric stabilization mechanism [11,12].

On the other hand, both emulsions and CNPs can be used as carriers of bioactive compounds, being used in the development of functional foods [13]. Bioactive molecules can modify the behavior of NPs in the emulsion, so studying each one of them is necessary. In addition, encapsulation in nanocarriers allows the masking of anomalous flavors and odors [14].

Protein hydrolysates and peptides are bioactive molecules of great interest due to the different biological activities that they manifest. In this sense, the fishing industry generates a large amount of protein waste that can constitute a source of high added-value co-products [15]. Fish protein hydrolysates (FPHs) are mainly produced by enzymatic hydrolysis, in which bioactive peptides with antioxidant [16,17], antimicrobial and anti-inflammatory [18] and anti-hypertensive [19] properties are obtained. In this context, the production of bioactive peptides from industrial by-products of stripped weakfish (*Cynoscion guatucupa*) by enzymatic hydrolysis was reported; the resultant hydrolysate showed antimicrobial and antioxidant activities, demonstrating its potential in food preservation applications [20] and a protective effect against oxidative stress in *Caenorhabditis elegans* [21].

Nanoparticles obtained by chitosan (C) and tripolyphosphate (TPP) have been largely studied as carriers of bioactive molecules due to their entrapment characteristics. However, several parameters can influence their production and encapsulation efficiency. The aim of this research was to develop chitosan-based NPs, with or without an encapsulated bioactive fish protein hydrolysate (FPH), and to evaluate their structural and bioactive properties, as well as their behavior in a food emulsion (mayonnaise type).

## 2. Materials and Methods

### 2.1. Materials 

The reagents and materials used were as follows: chitosan was purchased from Guinama (Valencia, Spain); the molecular weight (220 kDa) determined by a glass capillary viscometer and degree of deacetylation (78.6%) by Ftir determined from the ratio of absorbance: (A1655 cm^−1^/A3450 cm^−1^) × 100/1.33 were conducted according to Fernández-Martín et al. [22]. Sodium tripolyphosphate (TPP) 85% technical grade (Sigma-Aldrich, St. Louis, MO, USA); sunflower oil 0.2° (Sovena Spain S.A., Brenes, Seville, Spain); pasteurized liquid egg white (Huevos Guillén S. L., Valencia, Spain) and Protamex from Sigma-Aldrich (St Louis, MO, USA) were obtained. Stripped weakfish (*Cynoscion guatucupa*) by-products were obtained from the fishery industry in the city of Rio Grande, RS, Brazil. The muscle from the carcasses and trimmings was obtained after processing in a meat–bone separator (High Tech, HT250C), which separated the meat from the skin and bones.

### 2.2. Synthesis of Chitosan Nanoparticles

Chitosan nanoparticles (CNPs) were prepared by the process of ionic gelation as described by Piras et al. [23] and Hosseini et al. [24], with modifications. Initially, CNP elaboration was conducted using different concentrations of chitosan, ultra-sonication and different chitosan/acetic acid/TPP ratios (Table 1 and Table 2) in order to determine the synthesis with better properties of size, polydispersity index and zeta potential. From these preliminary analyses, the synthesis with 3:1:1 and 3:3:3 weight ratio of chitosan mg/mL/acetic acid %/TPP mg/mL were selected for the encapsulation test. In their preparation, chitosan was solubilized in aqueous acetic acid (1% or 3% *v*/*v*) at a concentration of 3 mg/mL, remaining under stirring overnight. Subsequently, the pH was adjusted to 5. To produce the nanoparticles, 10 mL of TPP (1 mg/mL or 3 mg/mL) was added dropwise to 25 mL of the chitosan solution under constant stirring. After adding TPP, the suspension was stirred for 30 min at 750 rpm (MM30E, Ovan, Badalona, Spain). 

The CNPs were also loaded with a protein hydrolysate from stripped weakfish (*Cynoscion guatucupa*). The protein hydrolysate was obtained using the enzyme Protamex with a degree of hydrolysis (DH) of 5% according to Lima et al. [21]. Briefly, the recovered muscle was homogenized with distilled water (10% *w*/*v*, protein/distilled water), followed by inactivation of endogenous enzymes (85 °C for 10 min). The homogenate was hydrolyzed by Protamex (2% *w*/*w*; enzyme/protein) under optimal conditions (pH 7, 0; 50 °C). Hydrolysis was monitored by the pH-stat method and after reaching a hydrolysis degree of 5%, the enzyme was inactivated (90 °C for 10 min) with subsequent centrifugation where the supernatant was collected and freeze-dried. There was a protein concentration of 91 mg/100 mg and the presence of glutamic acid +glutamine (Glx), aspartic acid +asparagine (Asx), Alanine (Ala), and Glycine (Gly) were the most abundant amino acid residues in its composition and around 35–36% of hydrophobic residues [21]. The properties of this hydrolysate were previously described by Lima et al. [20,21].

After chitosan solubilization and pH adjustment, 20 mg of hydrolysate were added and homogenized in 25 mL of the chitosan solution, before the addition of TPP. The chitosan nanoparticles that did not contain the hydrolysate were labeled as CNP (synthesis 3:1:1 weight ratio), CNP3 (3:3:3 weight ratio) and those containing the hydrolysate CNPH or CNP3H, respectively. The suspensions were subjected to a sonication step at 50% amplitude for 4 min (cycles of 1 min and 1 min of stop) using a Q700 sonicator (Qsonica, Newton, CT, EEUU, max 700 W).

### 2.3. Nanoparticle Characterization

The average size of the nanoparticles (z-average) and the polydispersity index (PDI) were determined by dynamic light scattering and the ζ potential by Doppler laser electrophoresis, using a Zetasizer Nano SZ90 (Malver Instruments, UK) at 25 ± 0.1 °C. For this, the colloidal dispersion of CNPs were diluted in distilled water (10-fold) and the Brix degrees were determined. Determinations were performed in triplicate.

### 2.4. Entrapment Efficiency

The amount of entrapped hydrolysate was calculated as the difference between the total hydrolysate used for the preparation of CNPH and CNP3H and the non-trapped hydrolysate that was quantified by the Lowry method [25]. The non-trapped hydrolysate was separated from the nanoparticles using a 10 kDa membrane filter (Amicon® Ultra-15, Merck Millipore Ltd., Ireland) and centrifuged at 5000× *g* for 40 min at 4 °C (Multifuge 3 LR, Heraeus, Madrid, Spain). This procedure was also performed in the nanoparticles without hydrolysate (CNP and CNP3) as a blank. The entrapment efficiency (EE) of the hydrolysate in the NPs was calculated in triplicate according to Equation (1):

(1) $$\mathrm{EE}\;\left(\%\right)=\frac{(\mathrm{Total}\;\mathrm{hydrolysate}\;-\;\mathrm{filtrated}\;\mathrm{hydrolysate})\;}{\mathrm{Total}\;\mathrm{hydrolysate}\;}\;\times \;100$$

### 2.5. ABTS Radical Scavenging Activity 

2,2’-azinobis-3-ethylbenzothiazoline-6-sulfonic acid (ABTS) radical scavenging activity was determined according to Zheng et al. [26] with modifications. The ABTS radical was generated by incubating ABTS (7 mM) with potassium persulfate (140 mM) in the dark for 16 h at room temperature. In this test, the reaction was performed at 30 °C for 6, 15, 30, 45, and 60 min in the dark and absorbance at 734 nm was measured in a microplate reader. The test was carried out in triplicate.

### 2.6. Antimicrobial Activity

The antimicrobial activity of the CNPs was determined against *Staphylococcus aureus* CECT 240, *Listeria monocytogenes* CECT 4032, *Shigella sonnei* CECT 4887, *Escherichia coli* CECT 515, *Aeromona hydrophila* CECT 839T obtained from the Spanish-type culture collection (CECT). The screening susceptibility test was conducted by the disc diffusion method on solid Brain Heart agar (Merck Company, Darmstadt, Germany). Bacteria isolates were obtained from overnight cultures in Brain Heart agar and suspensions were prepared in sterile saline solution by adjusting the turbidity to match 0.5 McFarland standards. Then, sterile filter paper discs (5 mm in diameter) (Oxoid, England) soaked with 40 µL of CNP, CNPH, CNP3 and CNP3H were placed on the agar plates seeded with the respective bacteria and incubated at 37 °C for 24 h. The surrounding clear areas were considered as a measurement of the antimicrobial activity and were expressed as the mean of the inhibition zones (mm). All measurements were determined at least in triplicate.

### 2.7. ACE-Inhibitory Capacity

The capacity of the nanoparticles to inhibit angiotensin I-converting enzyme (ACE) was determined by liquid chromatography (RP-HPLC) according to Alemán et al. [27], with some modifications. The reaction was composed of 50 µL of 5 mM Hippuryl-L-Histidyl-L-Leucine (HHL), 80 µL of ACE (0.025 U/mL) and 20 µL of samples and after incubation; the reaction was stopped by adding 80 µL of HCl (0.1 M). Then, 50 µL of each sample was injected in a RP-HPLC system (Shimadzu SPE-MA10AVP, Kyoto, Japan) using a flow rate of 0.8 mL/min and acetonitrile gradient from 20% to 60% in 0.1% (*v*/*v*) of trifluoroacetic acid in 26 min. The results were expressed as a percentage (%) of ACE-inhibitory activity.

### 2.8. Morphology

The morphology of the CNPs was observed by transmission electron microscopy (TEM) using a Jeol microscope, JEM-1400 with an acceleration voltage of 100 kV. For this, the dispersions of the CNPs were concentrated 3-fold in a rotary evaporator (Büchi Rotavapor R-300 System, Tulln, Austria) using a temperature of 55 °C. Before the analysis, the samples were submitted to an ultrasound bath for 15 min and 1 or 2 drops were deposited in a 200-mesh copper grid, which was suspended for 10 min, followed by the removal of the excess sample. The grid remained in a desiccator for at least 24 h.

### 2.9. Application in a Food Emulsion Model 

Food emulsion systems were prepared using sunflower oil, the NPs and pasteurized liquid egg white (mayonnaise type) to determine if the NPs have some effect in the stability of this food emulsion model. The CNP and CNPH suspensions were 3-fold concentrated in a rotary evaporator (Büchi Rotavapor R-300 System, Tulln, Austria) before use. The emulsions were prepared using a 4:1.2:1 volume ratio of oil, egg white, and CNP or CNPH, respectively. For the control, a solution of acetic acid (1%, *v*/*v*; pH 5.0) was used instead CNP or CNPH. Then, the resulting mixtures were homogenized in an ultra turrax (model T50, IKA, IKA®-Werke GmbH & CO. Staufen, Germany) at 7000 rpm for 5 min.

#### 2.9.1. Stability and Lipid Release

The physical stability of this food emulsion model was evaluated by the centrifugation method, according to Kasiri and Fathi [28] and Barkhordari and Fathi [29], with some modifications. Briefly, the emulsions were centrifuged at 2500× *g* for 5 min at room temperature to accelerate phase separation by generating an oil phase at the top, an emulsion phase in the middle and an aqueous phase at the bottom. The initial weight of the emulsion (g) and the weight of the remaining emulsion after centrifugation (g) were evaluated. Emulsion stability (ES) was obtained according to Equation (2):

(2) $$\mathrm{ES}\;\left(\%\right)=\frac{\mathrm{weight}\;\mathrm{of}\;\mathrm{the}\;\mathrm{emulsion}\;\mathrm{remaining}\;\mathrm{after}\;\mathrm{centrifugation}\;}{\mathrm{initial}\;\mathrm{weight}\;\mathrm{of}\;\mathrm{the}\;\mathrm{emulsion}}\;\times \;100$$

Additionally, the % lipid release was determined by the weight of the oil in the supernatant (g) after centrifugation and the initial amount of oil used in the preparation (g) according to Equation (3):

(3) $$\mathrm{Lipid}\;\mathrm{release}\;\left(\%\right)=\frac{\mathrm{oil}\;\mathrm{in}\;\mathrm{supernatant}\;}{\;\mathrm{total}\;\mathrm{oil}\;\mathrm{used}\;\mathrm{in}\;\mathrm{preparation}\;}\;\times \;100$$

#### 2.9.2. Rheological Analyses

Oscillatory shear determinations were made on a Bohlin CVO rheometer (Bohlin Instruments Ltd., Gloucestershire, UK) using a cone-plate geometry (4° angle, 40 mm diameter, 0.15 mm gap). All tests were performed in triplicate at a controlled temperature of 25 °C. Frequency sweep tests were carried out over a range of angular frequencies between 0.63 and 63 rad/s with an oscillation strain of 5%, selected from the linear viscoelastic region (LVER). The storage modulus (G′; Pa) and loss modulus (G″; Pa) were registered as a function of angular frequency (ω). The power law model was used to fit both G′ (G′ = G′0·ωn′) and G″ (G″ = G″0·ωn″), where G0’ and G0″ are the respective moduli (storage and loss) at 1 rad/s; and n′ and n″ exponents denote the viscoelastic response in terms of the time stability of both G′ and G″ at short time scales. 

### 2.10. Statistical Analysis

The data were submitted to analysis of variance (ANOVA) and the means were compared by the Tukey test at a 5% level of significance using STATISTICA 7 Software.

## 3. Results and discussion

### 3.1. Size, PDI and Zeta Potential

Average particle size, PDI and zeta potential are important parameters to evaluate the properties of nanoparticles [30]. In acidic conditions, chitosan is positively charged due to the protonation of amino groups, which can interact with anions such as TPP by the ionotropic gelation technique, leading to the formation of CNPs [24,31]. For this reason, a preliminary study was performed using different concentrations of chitosan and ultrasonication in order to select the optimal concentration. The results from these initial tests (Table 1) showed that a short sonication time (2 min) and ultrasonic yield power (50%) caused a reduction in size and PDI when a chitosan concentration of 1 mg/mL was used; however, a reduction in the zeta potential also occurred. Furthermore, size and zeta potential were observed to increase with increasing chitosan concentration. From this, to obtain stable z potential [32] and low-polydisperse particles, a chitosan concentration of 3 mg/mL was selected due to its intermediate size, PDI < 0.3 and zeta potential of 40 mV, resulting in medium-sized particles. 

Subsequently, different concentrations of acetic acid and TPP were studied in order to select the best conditions for NP preparation using ultrasonic yield power (50%) for 4 min (cycles of 1 min and 1 min of stop) (Table 2). In preliminary works, higher amplitudes (75%) drastically increased particle size and PDI, while for times between 1–4 min, no marked changes were observed (data not shown). Therefore, 50% and 2 min of ultrasonication were selected. The samples with weight ratios of 3:1:1 (CNP) and 3:3:3 (CNP3) of chitosan, acetic acid and TPP, respectively, were selected for hydrolysate encapsulation; CNP was selected for its small size, greater zeta potential and PDI below 0.3, while CNP3 was selected for its lower PDI (Table 2).

The characteristics of the loaded and unloaded NPs are summarized in Table 2. CNPH and CNP presented no differences in size, zeta potential and PDI, and the same was observed for CNP3 and CNP3H. However, CNP3 and CNP3H nanoparticles showed significantly (*p* < 0.05) higher values for size and lower values for PDI and zeta potential, as compared with CNPH and CNP (Table 2). 

In the case of chitosan nanoparticles, the initial size of the nanoparticles depends on many factors including preparation (ultrasonic yield power, sonication time and stirring speed), temperature and pH of the solution, chitosan concentration, loaded drug concentration, chitosan to TPP ratio and so on. Some of these factors were considered in our previous tests (Table 1 and Table 2). However, according to the factors evaluated in this study, at the same pH, peptide composition and ultra-sonication conditions, increased concentrations of C produce larger NPs; this is related to the increase in -NH3^+^ that produce a stronger intramolecular repulsion, which causes the C chain to stretch and results in larger NPs [31]. In addition, it was observed that increasing the concentration of TPP reduces the zeta potential of the NPs, which is due to increased crosslinking, which results in the neutralization of the protonated amino groups via TPP anions. Similar results were reported in other works [33,34]. 

The results showed that the incorporation of the FPH does not affect the physical characteristics of the NPs as compared with the control, indicating that the FPH negatively charged molecules were able to crosslink with chitosan, producing homogenous nanoparticles with low polydispersity. Achieving minimum polydispersity is important to keep the protein release rate constant and controllable, considering that in the case of chitosan, it is not easy to obtain a narrow distribution due to the fact that its structure contains low, medium and high molecular weight chains [34]. In addition, zeta potential values around 38 mV and 26 mV in CNPH and CNP3H nanoparticles, respectively, were obtained (Table 2). The zeta potential determines the stability of colloidal systems, and nanoparticles are considered stable in suspensions with a potential greater than 30 mV [32]; thus, CNPH nanoparticles present good stability since their zeta potential is around +30 mV.

The entrapment efficiency (EE) values of CNPH and CNP3H were 59.6% and 56.0%, respectively; these values were lower than those obtained in previous reports of bovine serum albumin (BSA) encapsulated in chitosan NPs, namely 64% [34] and 72.5% [35]. Gan and Wang [35] reported an increase in the EE from 38.7 to 72.5% with increasing protein (BSA) concentration, since protein adsorption and encapsulation by electrostatic interactions normally exhibit saturation kinetics, reaching a peak value at the highest concentration tested (1.50 mg/mL). Thus, despite the differences in composition and molecular size between BSA and the FPH used in the present study, the lower EE obtained in this work could be related to the concentration of the FPH (0.57 mg/mL) used. In addition, there are some other factors that affect the EE of proteins in the chitosan NPs, such as peptide molecular weight and composition, pH of the solution and chitosan and TPP concentrations [35,36].

### 3.2. ABTS Radical Scavenging Activity

**Figure 1:**
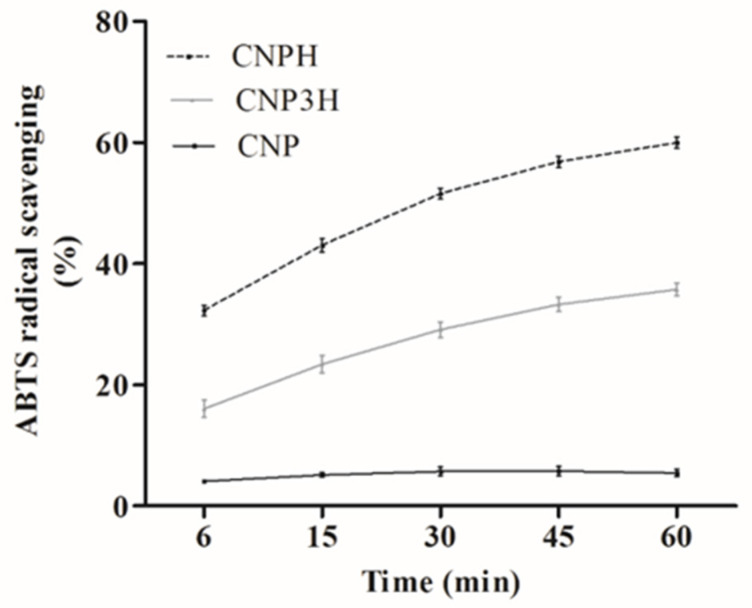
ABTS radical scavenging activity of nanoparticles over time.

The ABTS radical scavenging assay to assess the antioxidant capacity of peptides is usually conducted using an incubation period of 6 to 10 min. However, longer incubation times may be necessary to reach stable end-points [26]. The ABTS radical scavenging assay was performed at different incubation times in order to investigate the behavior of radical scavenging activity of the FPH in chitosan NPs (Figure 1). 

The results showed that the unloaded nanoparticles (CNP) presented a low ABTS radical scavenging activity and remained practically in the same range over time, while CNP3 showed no measurable ABTS radical scavenging activity. Chitosan can present radical scavenging capacity due to the amino and hydroxyl groups that react with free radicals [37,38]. However, some of the amino groups of C react with the crosslinking agent (TPP), resulting in the loss of antioxidant activity [39]. Thus, the use of acetic acid (3%, *v*/*v*) and TPP (3 mg/mL) in CNP3 possibly resulted in more occupied active sites, decreasing the availability of reactive groups and consequently influencing the antioxidant activity. In this context, Hadidi et al. [40] demonstrated a low DPPH radical scavenging activity for unloaded chitosan nanoparticles, which remained in a similar range at different concentrations.

In contrast, when the nanoparticles were loaded with the stripped weakfish hydrolysate (CNPH or CNP3H), a higher ABTS radical scavenging activity was observed. In a previous study, Lima et al. [20] reported ABTS radical scavenging activity for a stripped weakfish (*Cynoscion guatucupa*) hydrolysate. The hydrolysate alone showed no variations in ABTS radical scavenging activity over time when in contact with the radical (data not shown). In addition, in the present study a gradual increase in activity over time with CNPH was observed. The difference in activity may be related to the diffusion of peptides from the nanoparticles, suggesting that in CNP3H, most of the peptides with antioxidant activity remain inside the nanoparticles, and thus not interacting with the radical.

The increasing values of ABTS radical scavenging activity over time observed for CNPH and CNP3H suggest that the peptides with antioxidant activity present in the hydrolysate were gradually released from the CNPs according to the incubation time with the radical, showing a difference depending on the composition of the nanoparticles. Alamdaran et al. [41] reported that chitosan nanoparticles loaded with peptides derived from HIV-1 protein P24, obtained by ion gelation using TPP, present a release profile characterized by a rapid initial burst release in the first 24 h, followed by a slow constant release up to hour 192, demonstrating their potential as vehicles for a controlled and sustained release of peptide drugs.

Although composition appears to be an important factor, the efficiency of NPs as a delivery system for therapeutic agents depends on others factors such as NP size, hydrophobicity, and surface charge [42].

### 3.3. Antibacterial Activity

To determine the antibacterial activity against food pathogens such as *S. sonnei, S. aureus, A. hydrophila, E. coli* and *L. monocytogenes*, the agar disc diffusion method was used. As shown in Table 3, CNP presented activity only against *S. sonnei* and *A. hydrophila*, with inhibition zones of 6.37 and 9.69 mm, respectively. For CNPH, the highest inhibition halo was obtained against *S. aureus* (18.6 mm), but activity against *S. sonnei* and *A. hydrophila* (Table 3) was also observed. In the case of CNP3 and CNP3H nanoparticles, they also showed activity against *S. sonnei* and *S. aureus*, but the values of the inhibition halo were lower as compared with CNPH. However, no activity was observed against *E. coli* or *L. monocytogenes* in any of the samples. 

Chitosan nanoparticles present a broad spectrum of antibacterial activity, but they manifest different inhibitory efficacy against Gram-negative (G−) and Gram-positive (G+) bacteria. Moreover, the antibacterial activity of chitosan nanoparticles is triggered by several factors, including bacterial target Gram-negative vs. Gram-positive bacteria, growth stage, zeta potential, concentration, pH, molecular weight, and degree of acetylation [5].

The results revealed that the FPH at a concentration of 0.57 mg/mL enhanced the antimicrobial activity of CNP, increasing its antimicrobial activity, especially against *S. aureus* and *S. sonnei* (*p* < 0.05). In a previous work, a fish hydrolysate obtained from stripped weakfish (*Cynoscion guatucupa*) showed weak antimicrobial properties against *S. aureus* and *E. coli,* probably due to its composition of peptides with the presence of hydrophobic amino acids, which are responsible for acting on the wall of microorganisms [20]. Thus, the presence of hydrophobic amino acid residues on the CNPH surface increases their interaction with the bacterial cell surface, enhancing the modification of the permeability of the membrane, and therefore producing the osmotic imbalance and efflux of intracellular substances caused by CNP [43]. 

Due to the better results observed in the antimicrobial and antioxidant tests and to the physical properties presented, the study proceeded focusing only on CNPH and CNP.

### 3.4. ACE-Inhibitory Activity of Nanoparticles

**Figure 2:**
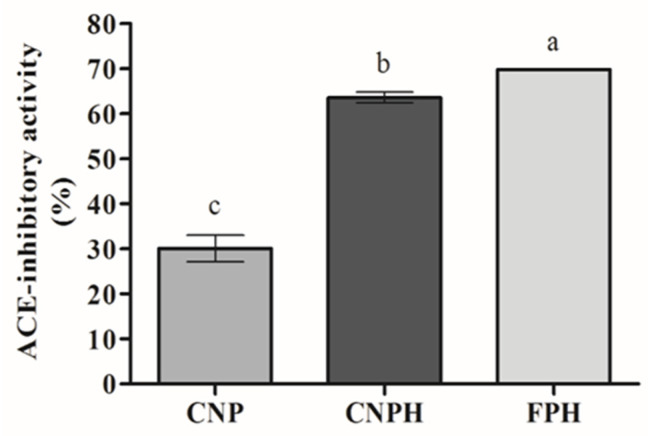
ACE-inhibitory activity of nanoparticles and fish protein hydrolysate (FPH). Different lowercase letters (a,b,c) indicate a significant difference between the samples (*p* < 0.05).

The ACE- inhibitory activity of the NPs is shown in Figure 2. CNPH showed an ACE-inhibitory activity around 2-fold higher than CNP (*p* < 0.05). The FPH (at 0.57 mg/mL) and CNPH presented similar activities, although a higher activity for CNPH was expected since CNP also presented ACE-inhibitory activity. However, the difference found between the FPH and CNPH can be attributed to the retention of some bioactive peptides in the nanoparticle structure.

Previous studies have reported ACE-inhibitory activity for chitosan nanoparticles [44,45]. However, the mechanism involved in the ACE-inhibitory activity of polysaccharides is unknown [46]. It is well documented that peptides from FPH present the ability to inhibit ACE [21,47], being possibly responsible for the increase in the ACE-inhibitory activity observed. 

The use of chitosan nanoparticles as carriers of bioactive peptides can be useful to protect the peptides by promoting resistance to the enzymatic degradation and severe pH found in the gastrointestinal tract, to favor a controlled release and to increase their oral bioavailability [48]. In this context, Auwal et al. [49] reported an ACE-inhibitory activity of 28.06% for stone fish biopeptides (molecular mass < 10 kDa) loaded in chitosan nanoparticles: TPP (per 0.025 mg of solid biopeptides), while Danish et al. [50] reported an ACE-inhibitory activity of 83 ± 7% and 82 ± 5% for leucine-lysine-proline and isoleucine-proline-proline tri-peptides (10 µM concentration), respectively. Nevertheless, these studies did not evaluate the activity of chitosan nanoparticles alone.

### 3.5. Morphology of Nanoparticles

**Figure 3:**
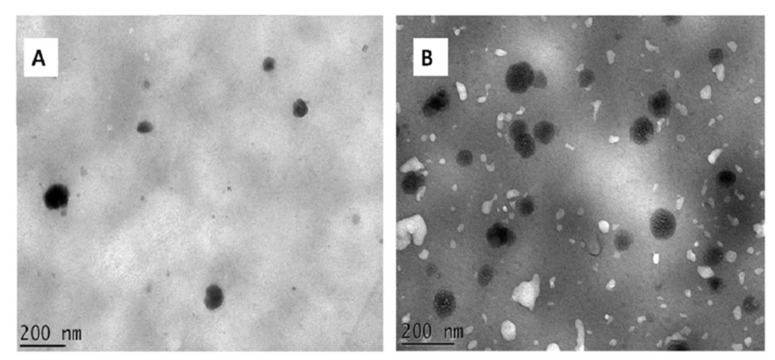
Transmission electron microscopy images of CNP (**A**) and CNPH (**B**). Bar = 200 nm.

The structure of the unloaded (CNP) and loaded (CNPH) nanoparticles were analyzed by TEM (Figure 3A,B). The morphology of CNP is smooth and presents a spherical shape (Figure 3A) in accordance with Pan et al. [32]. However, the sample CNPH showed structural differences inside of each nanoparticle (Figure 3B), which could indicate that the FPH was well integrated within the chitosan network. Similar morphological characteristics were demonstrated by Zhao et al. [51] for chitosan/TPP nanoparticles loaded with fish-derived peptides using chitosan with a molecular weight of 150 kDa and a 90–95% degree of deacetylation.

TEM micrographs showed a mean particle size of around 100 nm, which was smaller than the particle size measured by DLS; this size reduction relative to the fully hydrated samples was due to the TEM preparation procedure of the samples, which resulted in shrinkage, as CNPH and CNP were dried directly over the cooper grid [24].

### 3.6. Food Emulsion Model System 

**Figure 4:**
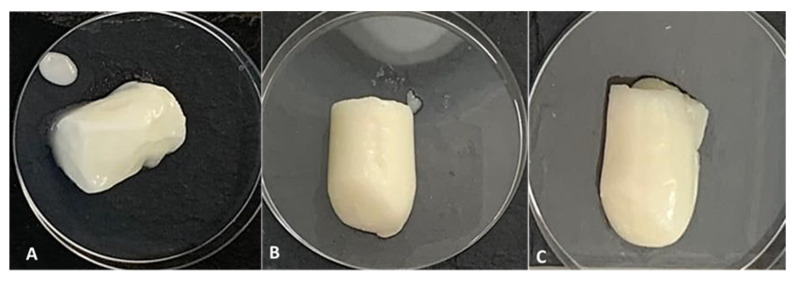
Visual appearance of the prepared control (**A**), CNP (**B**) and CNPH (**C**) food emulsions.

In the food industry, surfactants, hydrocolloids, or proteins are generally used as emulsion stabilizers, with the proteins being adsorbed at the oil–water interface forming a protective viscoelastic layer around the droplets [6]. The chitosan nanoparticles, empty or loaded with the fish protein hydrolysate, were applied in a food emulsion containing egg white in order to evaluate their application in a food emulsion model, with the possibility of being enhanced with biological activities, as shown above. The visual appearance of the prepared food emulsions is shown in Figure 4. The appearance was that of a very consistent mayonnaise-type emulsion for the formulas containing NPs, while the control was quite soft, showing the binding and pickering abilities of the nanoparticles (unloaded or loaded).

**Figure 5:**
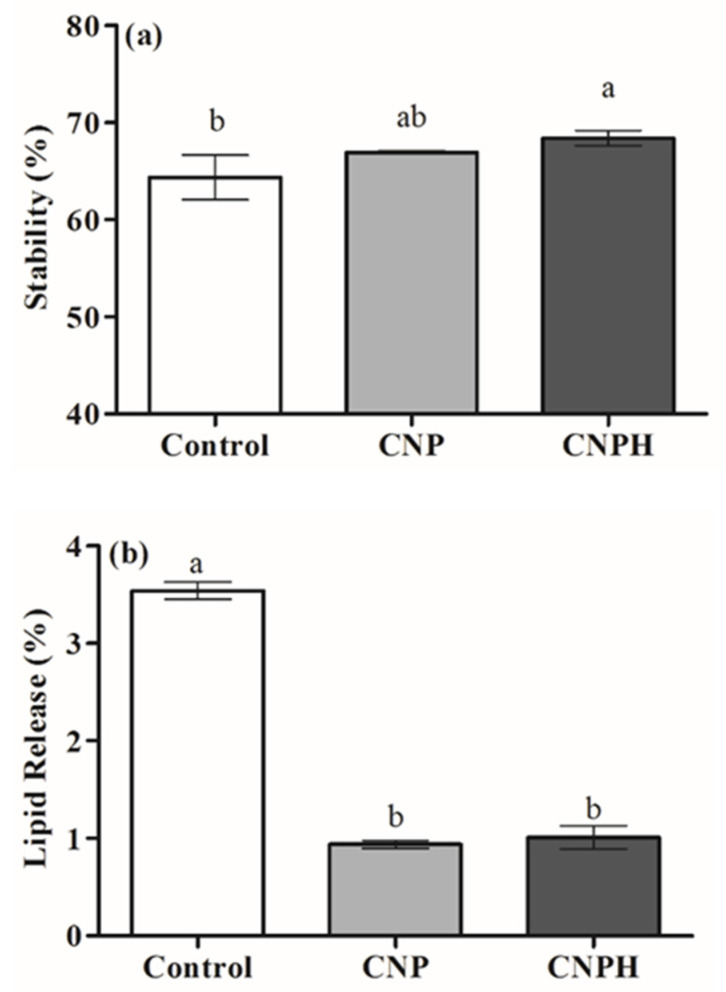
Emulsion stability (**a**) and lipid release (**b**) of the food emulsion. Different lowercase letters (a,b) indicate a significant difference (*p* < 0.05).

The effect of the chitosan nanoparticles on the stability of the emulsion (Figure 5a) and lipid release (Figure 5b) was investigated. As shown in Figure 5a, and compared with the control emulsion, the presence of the CNP dispersion did not have a negative effect on the stability of the food emulsion model; on the contrary, CNPH provided a small increase in stability (*p* < 0.05). 

The stability of the emulsion by the centrifugation method was based on a three-phase separation, with the formation of an oil phase, an emulsion phase and an aqueous phase at the top, middle and bottom, respectively. Similar stability results (around 65%) using the centrifugation method were observed by Barkhordari and Fathi [29] when using 1% *w*/*v* of chitin nanocrystals to produce a pickering emulsion simulating mayonnaise. In the case of chitosan-based nanoparticles used as emulsion stabilizers, several factors influence the stabilizing properties, such as wettability (contact angle), particle size and surface charge, the use of ultrasound, particle concentration and pH, among others [52].

In addition to stability, lipid release was evaluated as a reduction in the lipid release was demonstrated in the food emulsion model systems containing nanoparticles in comparison with the control (Figure 5b). This fact could also be verified visually, as shown in Figure 4, where the largest lipid exudation was visible in the control food emulsion. This result may be related to the adsorption of particles at the oil/water interface of the emulsion droplets [52], which, consequently, results in less oil release. In a previous study, Ribeiro et al. [53] demonstrated that chitosan-TPP nanoparticles are adsorbed at the interface, stabilizing the oil droplets, where ionic crosslinking leads to the formation of individual particles that concentrate on the droplet surface.

#### Rheological Properties

**Figure 6:**
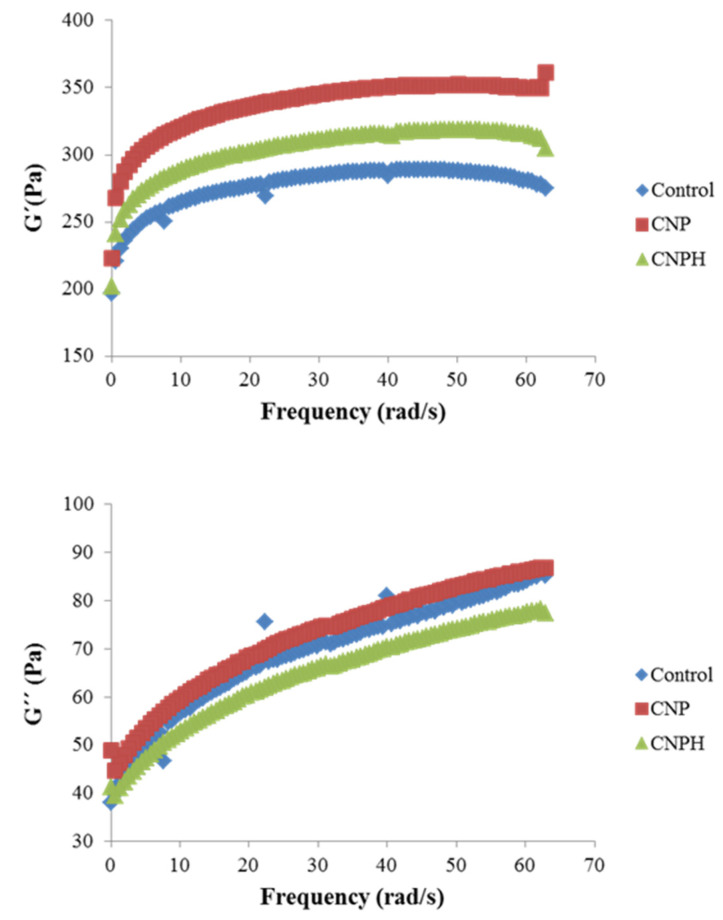
Mechanical spectra of emulsions.

The viscoelastic properties of the samples were analyzed with small amplitude oscillatory shear tests. The mechanical spectra of the three mayonnaise samples reveal a gel-like behavior, with predominance of G′ > G″ in the frequency range tested (Figure 6). Both the elastic and dynamic loss moduli increase with frequency, which indicates that the emulsion shows a certain level of structure, behaving as a gel-like solid [54]. The sample CNP presents the highest G′ values in the entire spectrum of frequencies analyzed, which suggests the reinforcing role of the nanoparticles in the network, probably through hydrogen bonds and electrostatic interactions between the protein and the nanoparticles, which in turn, lead to lipid entanglement. The elastic modulus values of the emulsion containing charged nanoparticles (CNPH) showed slightly lower values, which can be attributed to the predominant presence of negative charges in the hydrolysate, and therefore could cause an increase in repulsive electrostatic forces in the micellar structure [55]. The viscoelastic parameters G_0_′ and G_0_″ reflect the total resistance to deformation (elastic and viscous), thus constituting a measure of the gel resistance of the samples [56]. 

As can be seen in Table 4, G_0_′ values increase with the presence of the NPs, more so when the particles are not charged, which is consistent with what is observed in Figure 6. However, the loss modulus shows closer values between samples, with no differences between the control and CNP (*p* < 0.05). The greater differences between G′ and G″ in the samples with NPs can be seen in the loss factor (tang δ) (Table 4), suggesting a greater strength of intermolecular interactions and consequently greater stability of the gel [57] with the presence of the nanoparticles. Some authors have observed a similar behavior when making emulsions with nanoparticles. Chang et al. [58], in mayonnaise made with nanoparticles of egg white protein and pectin, also detected a gel-like behavior with increasing frequency, although at higher frequencies, close to 50 Hz, the loss modulus reached similar values to those of the elastic modulus, acquiring liquid-like properties. They also observed that mayonnaise had a higher G′ as the presence of nanoparticles was greater. Huang et al. [59] elaborated mayonnaise with CNPs, both unloaded and loaded with caseinophosphopeptide, observing that the addition of peptides decreased the viscoelastic properties, G′ and G″. However, the behavior observed in this work suggests that chitosan NPs may form block structures that contribute to the rigidity or mechanical strength of the pickering emulsions, while the emulsions with loaded NPs produce nanocomplexes that can be more efficiently adsorbed at the oil–water interface.

In the present work, it can be observed that tang δ values are quite high, which is indicative of a tendency to present a semi-liquid state and favors the viscous component, suggesting that the gel formed is very weak, being weaker in the case of the control sample. In fact, it can be observed that G″ increases notably with increasing frequency, although the values do not come close to, nor do they cross G′, as occurred in the mayonnaise, prepared by Chang et al. [58]. This fact is evidenced by the exponents n′ and n″, as n″ > n′, which means that the rate of decline of G″ with the reduction in ω was higher than that of G′, resulting in a shear-induced gelation at lower frequencies, i.e., at higher oscillation times. This effect was again more noticeable in the control sample without nanoparticles. According to Moreno et al. [60], this fact indicates a shear-induced enlargement in the energy stabilization of matrix bonds at lower frequencies, which is compatible with the decrease in gel strength at higher times of oscillation.

## 4. Conclusions

In this work, different chitosan nanoparticles (CNPs) were prepared by the ionic gelation technique with tripolyphosphate (TPP) and loaded with a fish protein hydrolysate (FPH). Due to the results of the CNP physical characterization, the samples with weight ratios 3:1:1 and 3:3:3 of chitosan, acetic acid and TPP, respectively, were selected for the fish protein hydrolysate (FPH) encapsulation. The ABTS radical scavenging activity of the NPs increased when they were loaded with the FPH, which was released over time, exhibiting noticeable antimicrobial activity against *S. aureus* and *A. hydrophila* and ACE-inhibitory activity, while the unloaded NPs did not show ABTS radical scavenging activity and presented lower antimicrobial and ACE-inhibitory activities. When added to a food emulsion model, the CNPs loaded with the FPH improved the stability, and both unloaded and loaded NPs decreased the lipid release. Thus, CNPs were able to transport the FPH and exhibited antioxidant, antimicrobial and ACE-inhibitory properties, being an alternative peptide nanocarrier with potential as an emulsion stabilizer for food applications.

## Figures and Tables

**Table 1:** Initial tests with different concentrations of chitosan nanoparticles prepared with acetic acid (1% *v*/*v*) and TPP (1 mg/mL).

Chitosan (mg/mL)	Ultrasound/Time	Size	PdI	Zeta
1	-	342.6 ± 1.1 ^c^	0.279 ± 0.01 ^a^	20.5 ± 1.6 ^c^
1	50%/2 min	164.8 ± 0.3 ^d^	0.166 ± 0.02 ^b^	16.3 ± 0.9 ^d^
3	50%/2 min	314.5 ± 6.1 ^c^	0.254 ± 0.01 ^a^	40.0 ± 1.1 ^b^
7	50%/2 min	486.8 ± 14.0 ^b^	0.316 ± 0.04 ^a^	43.2 ± 1.0 ^a^
10	50%/2 min	601.9 ± 29.3 ^a^	0.264 ± 0.04 ^a^	42.1 ± 0.9 ^ab^

Different superscript letters (a,b,c,d) indicate significant differences within the same column (*p* < 0.05). Values are the means ± standard deviation of three independent experiments.

**Table 2:** Nanoparticle characterization.

Sample	Chitosan (mg/mL)	Acetic Acid (%)	TPP (mg/mL)	Size (nm)	PdI	ζ Potential (mV)
CNP	3	1	1	205.7 ± 6.5 ^bB^	0.281 ± 0.05 ^bA^	39.1 ± 1.6 ^aA^
CNP13	3	1	3	273.6 ± 1.5 ^a^	0.171 ± 0.03 ^c^	28.1 ± 0.6 ^b^
CNP3	3	3	3	264.3 ± 0.8 ^aA^	0.146 ± 0.01 ^cC^	27.3 ± 0.8 ^bB^
CNP31	3	3	1	199.8 ± 7.4 ^b^	0.371 ± 0.02 ^a^	37.1 ± 1.7 ^a^
CNPH *	3	1	1	202.2 ± 2.4 ^B^	0.237 ± 0.01 ^AB^	37.7 ± 1.2 ^A^
CNP3H *	3	3	3	263.1 ± 1.9 ^A^	0.169 ± 0.05 ^BC^	26.0 ± 0.9 ^B^

* Nanoparticles loaded with hydrolysate. Different lowercase letters (a,b,c) indicate significant differences within the same column of unloaded NP (*p* < 0.05). Different uppercase letters (A,B,C) indicate significant differences between CNP, CNP3, CNPH and CNP3H (*p* < 0.05).

**Table 3:** The mean of the diameters of the inhibition zones of CNP and CNPH against five bacteria.

Sample	Inhibition Zone (mm)				
	*S. sonnei*	*S. aureus*	*A. hydrophila*	*E. coli*	*L. monocytogenes*
CNP	6.37 ± 0.11 ^bB^	0.0 ± 0.0	9.69 ± 0.57 ^aAB^	0.0	0.0
CNPH	9.54 ± 1.01 ^bA^	18.6 ± 0.38 ^aA^	10.64 ± 1.14 ^bA^	0.0	0.0
CNP3	8.40 ± 0.49 ^bA^	11.75 ± 1.25 ^aB^	0.00 ± 0.0	0.0	0.0
CNP3H	9.12 ± 0.23 ^aA^	7.59 ± 0.24 ^bC^	8.17 ± 0.53 ^bB^	0.0	0.0

Different lowercase letters (a,b) indicate significant differences within the same line (*p* < 0.05). Different uppercase letters (A,B,C) indicate significant differences within the same column (*p* < 0.05).

**Table 4:** Viscoelastic parameters of emulsions.

	Control	CNP	CNPH
G_0_′ (Pa)	256.33 ± 4.94 ^c^	310.70 ± 12.10 ^a^	279.25 ± 3.69 ^b^
n′	0.0568 ± 0.004 ^a^	0.0637 ± 0.001 ^a^	0.0639 ± 0.003 ^a^
R (eq)	0.9278	0.9809	0.9744
G_0_″ (Pa)	52.15 ± 1.30 ^ab^	55.27 ± 2.84 ^a^	48.91 ± 1.02 ^b^
n″	0.1660 ± 0.007 ^a^	0.1441 ± 0.001 ^b^	0.1500 ± 0.002 ^b^
R (eq)	0.9109	0.8669	0.8804
tan δ	11.50 ± 0.06 ^a^	10.09 ± 0.13 ^b^	9.94 ± 0.08 ^b^

Different lowercase letters (a,b,c) indicate significant differences within the same line (*p* < 0.05).

## Data Availability

All data relevant to the study are included in the article.
